# Cadaveric Dissection a Thing of the Past? The Insight of Consultants, Fellows, and Residents

**DOI:** 10.7759/cureus.2418

**Published:** 2018-04-03

**Authors:** Haider Ghazanfar, Sannah Rashid, Ashraf Hussain, Madiha Ghazanfar, Ali Ghazanfar, Arshad Javaid

**Affiliations:** 1 Internal Medicine, Shifa College of Medicine, Islamabad, PAK; 2 Pediatrics, Shifa College of Medicine, Islamabad, PAK; 3 Department of Anatomy, Shifa College of Medicine, Islamabad, PAK; 4 Emergency Medicine, Shifa International Hospital, Islamabad, PAK; 5 Federal Medical and Dental College, Islamabad, PAK

**Keywords:** perception, anatomy, dissection, female, physician

## Abstract

Objective

The objective of our study was to determine whether cadaveric dissection is a necessity in medical education. Another purpose of our study was to assess the attitude and perception of consultants, residents, and fellows about cadaveric dissection and whether it helped them in their medical practices.

Method

We performed an analytical cross-sectional study among consultants, fellows, and residents of different specialty areas practicing in Punjab. A self-constructed questionnaire compromising of 41 items was used to assess the perception of doctors about cadaveric dissection and other alternative anatomy teaching methods. Consultants, fellows, and residents who were in clinical practice for more than six months were included in the study.

Results

Out of the total sample size of 842, 44.7% were female medical doctors and 55.3 % were male medical doctors. Cadaveric dissection was thought to be the most effective method for teaching anatomy by 27.9% of the doctors. Mean cadaveric dissection, prosection and didactic teaching components were scored significantly higher by doctors in surgery and allied fields (p<0.001). Doctors in the surgical and allied field were 0.55 times less likely to think that cadaveric dissection was unethical as compared to doctors working in medicine and allied fields (p<0.001).

Conclusion

Dissection is still considered by several doctors as a valuable source of learning anatomy. However, the future of teaching anatomy does not depend on any single method. It is, in fact, the right combination of all available resources and using them in an interactive way that maximizes outcomes.

## Introduction

Anatomy is derived from the Greek word “anatome” which means cutting up. It is the branch of science that studies the structure of the body. Over the past couples of decades, significant advances have occurred in the field of anatomy. Anatomy is one of the core basic science subjects studied by medicals student all across the world when they begin their medical education.

Traditionally, block system of education has been used in medical colleges to teach anatomy along with other core basic science subjects. Cadaveric dissection has been used for centuries for teaching gross anatomy all over the world [1]. Cadaveric dissection has been considered a necessity in the learning of gross anatomy and thought to contribute significantly to a future professional career [2-4]. Cadaveric dissection helps medical students by helping them in understanding the three-dimensional relationship of different anatomical structures and appreciating anatomical variations [5]. It has become a greatly acknowledged fact that good medical or surgical practice could only be based on adequate anatomical knowledge of human anatomy which can only be learned from cadaveric dissection [6].

Medical education's paradigm has shifted towards problem-based learning and integrated curriculum [7] This has resulted in the studying of core basic science subjects including anatomy in the light of clinical context only and the abandoning of cadaveric dissection. In most institutions, gross anatomy knowledge is now taught via a small group discussion based on various clinical cases which are followed by a whole class lecture session [8-9].

The paucity of cadavers and high financial cost have significantly contributed to the development of alternative teaching techniques; consequently leading to more medical students having no experience of cadaveric dissection. Furthermore, advances in web-based medical technology have resulted in the development of virtual dissection programs. These programs have been found to be an effective way teaching anatomy [10] and are being preferred over cadaveric dissection, as its use is not associated with emotional and ethical issues [11].

Alternative teaching methods are now more commonly deployed in teaching gross anatomy. This issue will become even more common with the passage of time. The age-old debate of ‘to dissect, or nor to dissect’ still rages on. The majority of medical students don’t do cadaveric dissection nowadays. Does this mean these medical students won’t become better doctors? Is cadaveric dissection simply a rite of passage or is it a necessity? The aim of our study was to determine whether cadaveric dissection is a necessity in medical education and can it be replaced by newer alternative techniques. Another purpose of our study was to assess the attitude and perception of consultants, residents, and fellows about cadaveric dissection and whether it helped them in their medical practices.

## Materials and methods

We performed an analytical cross-sectional study among consultants, fellows and residents of different specialties practicing in Rawalpindi, Islamabad, Wah, Multan, Bahawalpur, and Lahore from January 2016 to April 2017. A self-constructed questionnaire compromising of 41 items was used to assess the perception of consultants, fellows, and residents about cadaveric dissection and other alternative anatomy teaching methods. Consultants, fellows, and residents who were in clinical practice for more than six months were included in the study. Participants were chosen by stratified randomized sampling. The sample size of 802 was calculated by using the World Health Organization (WHO) sample size calculator, keeping 2.25 as absolute precision required, the prevalence of cadaveric dissection at 88%, and the confidence level at 95. A total of 1000 forms were given out of which 84.2% (842) were completely filled. Each participant was given 10 minutes to fill the form which was followed by a 10-minute interview. Informed consent was taken from all the participants and the identity of the respondents was kept anonymous. 

SPSS version 21 was used to analyze the data. After the responses of the consultants, fellows and residents were gathered, the internal validity of the questionnaire was tested using Cronbach’s alpha. The total questionnaire Cronbach’s alpha value was calculated as 0.712 providing evidence for the acceptable internal validity of the questionnaire.

Mean and the standard deviation was calculated for quantitative variables like age and the question addressing how well the teaching aim was achieved. Frequency and percentage were calculated for qualitative variables like gender, specialty type, and questions related to the perception of cadaveric dissection. Independent t-test was performed to find the association between specialty type and the question addressing the perception of the consultant. A p-value of less than 0.05 was considered as significant.

## Results

Mean age of the participants was 34.78±8.70. Out of the total sample size of 842; 44.7% (376) were female medical doctors and 55.3% (466) were male medical doctors. About 41.8% of the doctors were in surgery and allied and 58.2% of the doctors were in medicine and allied. About 411 (48.8%) of the doctors were working in private hospital while 431 (51.2%) of the doctors were working in a government hospital. About 349 (41.4%) of the participants were consultants while 493 (58.6%) of the participants were fellow and residents.

Cadaveric dissection was thought to be the most effective method for teaching anatomy by 27.9% (235) of the doctors. About 11.5% (97) doctors had not performed cadaveric dissection during the undergraduate program while 88.5% (745) had performed cadaveric dissection at least once during the undergraduate program. About 58.1% of the doctors were of the opinion that cadaveric dissection can be replaced by alternative teaching method. About 47.4% doctors were of the opinion that cadaveric dissection in an undergraduate program can help in their current specialty. Around 43.3% doctors disagreed with the statement that cadaveric dissection is must for every doctor. Only 3.3% doctors thought that cadaveric dissection is a waste of time. Around 47.5% doctors thought that cadaveric dissection is unethical. About 56.3% doctors believed that cadaveric dissection was religiously acceptable. Around 44.7% doctors thought that cadaveric dissection was worth the cost spent on it. 

About 64.0% doctors agreed that it was possible to practice their current specialty without doing cadaveric dissection in the undergraduate program. About 28.7 % doctors thought that cadaveric dissection helps in clearing post-graduate examinations. Around 28.7% doctor thought that cadaveric dissection helps a person in becoming a better doctor. Only 22.4% of the doctors thought that cadaveric dissection helps in diagnosing diseases. While Only 13.4% doctors were of the opinion that cadaveric dissection helps in deciding which specialty to opt for and only 8.8% doctors reported that cadaveric dissection helped them in attaining the current position.

The live surgeries method had the highest score in the method of teaching anatomy while didactic teaching method had the lowest score. Mean total score of each method of teaching anatomy has been shown in Table 1.

**Table 1:** Mean Total Score of Teaching Methods

Method of Teaching Anatomy	Total Mean±SD
Cadaveric Dissection	2.90±1.01
Cadaveric Prosection	2.78±0.91
Model	3.00±0.89
Computer Model Programs	2.86±1.00
Live Surgeries	3.44±0.73
Didactic Teaching	2.42±0.86

Independent sample t-test was applied to assess the association between type of specialty field and scores of different categories of teaching anatomy. Mean cadaveric dissection, prosection, and didactic teaching components were scored significantly higher by doctors in surgery and allied field while live surgeries component was scored significantly higher by doctors in medicine and allied field (p<0.001). This has been shown in Table 2.

**Table 2:** Association Between Type of Specialty and Scores of Different Categories of Teaching Anatomy

	Surgery and Allied	Medicine and Allied		
Method of Teaching Anatomy	Total Mean±SD	Total Mean±SD	t	p-value
Cadaveric Dissection	3.17±1.12	2.70±0.88	6.834	<0.001
Cadaveric Prosection	3.11±0.85	2.54±0.88	9.294	<0.001
Model	3.05±0.90	3.08±0.88	-0.564	0.573
Computer Model Programs	2.83±1.08	2.88±0.96	-0.646	0.518
Live Surgeries	3.26±0.64	3.57±0.77	-6.207	<0.001
Didactic Teaching	2.62±0.70	2.28±0.94	5.615	<0.001

Independent sample t-test was applied to assess the association between Type of hospital and scores of different categories of teaching anatomy. Mean cadaveric dissection, prosection, model, computer model programs, and didactic teaching components were scored significantly higher by doctors working in private hospitals while live surgeries component was scored significantly higher by doctors in public hospitals (p<0.001). This has been shown in Table 3.

**Table 3:** Association Between Type of Hospital and Scores of Different Categories of Teaching Anatomy

	Private Hospital	Public Hospital		
Method of Teaching Anatomy	Total Mean±SD	Total Mean±SD	t	p-value
Cadaveric Dissection	3.62±0.77	2.21±0.69	27.959	<0.001
Cadaveric Prosection	3.42±0.64	2.17±0.69	27.198	<0.001
Model	3.47±0.611	2.69±0.95	14.297	<0.001
Computer Model Programs	3.54±0.66	2.20±0.84	25.637	<0.001
Live Surgeries	3.34±0.80	3.53±0.65	-3.678	<0.001
Didactic Teaching	2.75±0.90	2.11±0.70	11.601	<0.001

Independent sample t-test was applied to assess the association between gender and scores of different categories of teaching anatomy. Mean cadaveric dissection, prosection and models were scored significantly higher by the female while computer model programs, Live surgeries, and Didactic teaching components were scored significantly higher by male doctors (p<0.05). This has been shown in Table 4.

**Table 4:** Association Between Gender and Scores of Different Categories of Teaching Anatomy

	Male	Female		
Method of Teaching Anatomy	Total Mean±SD	Total Mean±SD	t	p-value
Cadaveric Dissection	2.74±0.97	3.09±1.03	-4.999	<0.001
Cadaveric Prosection	2.58±0.89	3.03±0.88	-7.235	<0.001
Model	2.91±1.00	3.27±0.70	-5.807	<0.001
Computer Model Programs	2.96±1.08	2.74±0.90	3.195	0.001
Live Surgeries	3.55±0.62	3.30±0.83	5.226	<0.001
Didactic Teaching	2.56±1.03	2.25±0.55	5.262	<0.001

Pearson Correlation was done to assess for an association between age and scores of a different method of teaching anatomy. Pearson correlation showed significant positive correlation between age and cadaveric dissection method p<0.0005 (r=0.152), cadaveric prosection p<0.0005 (r=0.125), age Models p<0.0005 (r=0.318), computer model program p<0.005 (r=0621) and didactic teaching p<0.005 (r=0.405). Pearson correlation showed a negative correlation between age and live surgeries p=0.008 (r= -0.091).

Pearson chi-square test was applied to assess the association between gender, type of specialty and type of Hospital with “do you think Cadaveric Dissection is unethical”. Doctors in surgical and allied field were 0.55 times less likely to think that cadaveric dissection was unethical as compared to doctors working in medicine and allied field (p<0.001) while doctors working in private were 0.107 times less likely to think that cadaveric dissection was unethical as compared to doctors working in public hospital (p<0.001).

## Discussion

Anatomy in the past was considered as an important component of basic sciences in medical colleges and most of its teachings were based on detailed learning using cadaveric dissection. Being a very broad subject, it was soon realized that majority of its details were inapplicable and unnecessary in undergraduate programs because of poor long-term retention of information. This caused the conventional detailed curriculum to enfold into an applied knowledge form and the importance of anatomy as a major subject got reduced. Previously taught mainly using dissection augmented with didactic teaching and models [12], as the need of elaborate subject knowledge in clinical practice lessened, the use of dissection as its main mode of teaching was re-evaluated for benefits against high cost [13]. This led to the formation of two distinct schools of thoughts regarding the use of dissection as an anatomy teaching resource in medical colleges of Pakistan. The more financially equipped and government-sponsored teaching hospitals, due to ease of access to cadavers, continued using dissection as their main teaching tool augmented by prosection and didactic teaching; whereas, colleges funded privately discontinued it and replaced it with prosection, didactic teaching, models, computer-based programs and live surgeries. The purpose of this study was to assess the views of graduates from both college types regarding the better was of learning anatomy.

Students with access to dissection rated it to be less useful in comparison to computer-based programs and live surgeries. One reason could be the difficult student cadaver ratio of 50:1 that students have to counter in government-sponsored medical colleges along with the conditions of poorly ventilated, pungent smelling dissection halls [12]. According to our study, mean cadaveric dissection, prosection, model, computer model programs, and didactic teaching components were scored significantly higher by doctors working in private hospitals while live surgeries component was scored significantly higher by doctors in the public hospitals (p<0.001).

Previous studies concluded that learning anatomy was dependent more on memorization of organ systems and long lecture-based sessions with very little active student collaboration and applied experience involved, which led to most of the memorized knowledge acquired, lost by the time clinical experience began. However, in much accordance with previous study results [14], graduates largely believed that independent of the teaching resource used, instructors should make anatomy an interactive and involving subject with teamwork so the core concepts could be better understood and retained. A study also suggested that the end effect of a computer-based program was superior to the conventional ones used before and was also enjoyed by the students [15]. Incorporating lectures with role plays, animations, clinical cases in the form of problem-based learning result in active participation of students and hence may provide greater satisfaction and longer retention of core concepts in students. Although most methods (dissection and computer-based learning, live surgeries) have prevailed in medical colleges of Pakistan, neither were able to content students because the way these methods were used was similar - passive one-sided flow of information that usually resulted in a lack of interest in the subject and loss of concentration at the students end. Therefore, the preferred method chosen by doctors belonging to the two different college types was expecting it to be more interactive and fruitful, the one not experienced by them during their basic sciences years.

As the use of computer-based programs and related technologies as a teaching tool for anatomy rise around the world [16], its prospects of becoming a replacement for dissections in Pakistan was also addressed as part of this study and the response was analyzed in relation to gender. The result of this study also emphasized the global trend regarding the preferential use of technology, IT and software by male and female students in comparison to the conventional teaching methods. Our study showed that boys preferred the use of computer-based programs for educational purpose whereas girls favored conventional dissection as their choice of study tool. The reason for this difference was the greater accessibility of computers and software among boys as compared to girls in the country. Therefore, the ease of its use and operating confidence is higher in boys as compared to girls. Like most developing countries, access to computers and the internet is not easily available to students all around Pakistan. Therefore, similar to foreign study results, undergraduates in Pakistan also possess very limited knowledge of computers [17]. A study showed that the literacy level related to the use of the internet for accessing eHealth based information varied among female students [18]. Similar differences also prevail in Pakistan.

In our study, mean cadaveric dissection, prosection, and didactic teaching components were scored significantly higher by doctors in surgery and allied fields while live surgeries component was scored significantly higher by doctors in medicine and allied fields (p<0.001). A study conducted on Venezuelan surgeons concluded that 88% surgeon thought that cadaveric dissection was the most effective way to teach anatomy [19]. According to another study, 65% surgeons selected “cadaver/prosection demonstration” as the most effective way to teach anatomy [20]. One study suggested that full-body dissection should be reserved for medical students who intend to pursue a surgical career while prosections and plastination are more suitable for medical students who intend to pursue a career in the medical and allied fields [13].

According to various studies, younger physicians were less likely to prefer cadaveric dissection as compared to their seniors [19]. In our study, a significant positive correlation was seen between age and cadaveric dissection method p<0.0005 (r=0.152). Among other tools of learning anatomy, the ethical aspect of deforming a human body and hence violating its sanctity has been brought into question time and time again. The view of many clinical physicians has not changed regarding the ethical aspect of cadaveric dissection. The majority of the surgical and allied specialties thought that it is ethical practice to perform dissection on cadavers. They put cadaveric dissection as the main method of learning and supported its use in their own clinical decision making.

However, to consider cadaveric dissection as a tool for learning ethics still remains a matter of personal opinion and its perception varies from personal beliefs to the religious upbringing of an individual [21-22]. To add another dimension, the method of acquiring the bodies is also another process not properly regulated in many locations and leaves room for more questions as to whether cadaveric dissection should give way to more recent and innovative methods of learning.

## Conclusions

Dissection is still considered by several doctors as a valuable source of learning anatomy. However, the future of teaching anatomy does not depend on any single method. It is, in fact, the right combination of all available resources and using them in an interactive way that maximizes outcomes.
